# Drug Resistance Molecular Markers of *Plasmodium falciparum* and Severity of Malaria in Febrile Children in the Sentinel Site for Malaria Surveillance of Melen in Gabon: Additional Data from the Plasmodium Diversity Network African Network

**DOI:** 10.3390/tropicalmed8040184

**Published:** 2023-03-23

**Authors:** Jacques Mari Ndong Ngomo, Denise Patricia Mawili-Mboumba, Noé Patrick M’Bondoukwé, Bridy Moutombi Ditombi, Jeanne Vanessa Koumba Lengongo, Fanny Bertrande Batchy Ognagosso, Marielle Karine Bouyou-Akotet

**Affiliations:** Faculty of Medicine, Department of Parasitology and Mycology, Université des Sciences de la Santé, Libreville BP 4009, Gabon

**Keywords:** molecular markers, ART resistance, malaria clinical forms, parasitaemia, *Plasmodium falciparum*, Gabon

## Abstract

The objective of this study was to analyze the relationship between the frequency of artemisinin-based combination (ACT) drug resistance molecular markers and clinical forms of *P. falciparum* malaria and parasitemia. A cross-sectional study was carried out between January and April 2014 at the Operational Clinical Research Unit of Melen in febrile children aged 12 to 240 months with a *Plasmodium* sp. infection. A total of 3 mL of peripheral blood collected from an EDTA tube was used for leukocyte depletion. DNA mutation detection was performed by next generation sequencing (NGS). A total of 1075 patients were screened for malaria. Among them, 384 had a *Plasmodium* infection. *P. falciparum* mono-infection was found in 98.9% of the patients. *Pfcrt*-326T mutation was found in all isolates, while 37.9% had *Pfmdr2*-484I mutant allele. The highest median parasite densities were found in patients infected by parasites carrying the CVIET haplotype of the *Pfcrt* gene. The different genetic profiles found here, and their variations according to clinical and biological signs of severe malaria, are additional arguments for the surveillance of *P. falciparum* strains.

## 1. Introduction

Malaria cases were estimated at 247 million in the world in 2021. During this year, the number of malaria deaths was 619,000, of which 80% were children under the age of 5 [1]. *Plasmodium (P.) falciparum* is responsible for a severe form of the disease. Since the start of the 2000s, the World Health Organization (WHO) has recommended the use of sulfadoxine-pyrimethamine combination for preventive treatment against malaria during pregnancy, and artemisinin-based combinations (ACTs) for the treatment of uncomplicated malaria. The main combinations are artemether-lumefantrine (AL), artesunate-amodiaquine (AS-AQ), dihydroartemisinin piperaquine (DH-PQ), artesunate-mefloquine (AS-MQ) and artesunate-sulfadoxine-pyrimethamine (AS-SP). Severe malaria is treated with injectable artesunate (intramuscular or intravenous), followed by a complete three-day ACT course once the patient can tolerate oral medicines. Artemether and quinine may also be used in the absence of artesunate [2].

These recommendations have been adopted after the spread of *P. falciparum* strains multiresistant to conventional antimalarials, some of which are partner’s molecules of artemisinin derivatives in ACTs. ACTs led to a significant reduction in malaria morbidity and mortality in populations at risk [1,3]. Thus, WHO recommends regular monitoring of the efficacy of these antimalarial medicines to ensure that the chosen treatments are still efficacious.

In Southeast Asia, the resistance of parasites against artemisinin (ART) derivatives, as well as Piperaquine, has been shown [4]. Resistance to ART and its derivatives is characterized by an extension of the parasite clearance time in treated individuals [4,5]. Indeed, the persistence of parasites after three days of ACT treatment has been observed [5].

Moreover, the genetic background of ART resistant *P. falciparum* strains has been analyzed through a genome-wide association study [5]. Mutations Y493H, R539T, R561H, I543T and C580Y, located on the *P. falciparum klech 13* (*Pfk13*) gene in resistant parasites have been related to artemisinin resistance [6,7,8,9,10].

Other mutations have been found in other genes, such as *P. falciparum* ferrodoxine (Pffd- D193Y), *P. falciparum* apicoplast ribosomal precursor S10 (*Pfarps*-V127M), *P. falciparum* multidrug resistance protein 2 (*Pfmdr2*-T484I) and *P. falciparum* chloroquine resistance transporter (*Pfcrt*- I356T and *Pfcrt*- N326S) [11,12]. These mutations may contribute to the development of the parasite’s resistance to ART [13]. Likewise, the T484I mutation of *P. falciparum* multidrug resistance protein 2 (*Pfmdr2*) has been associated with artemisinin drug resistance [10,13]. Such resistance may be related to a higher risk of developing severe malaria linked to a change in the fitness of the *P. falciparum* strains.

Monitoring the resistance of malaria parasites to partner drugs is also necessary [14]. Two genes, *Pfcrt* and *Pfmdr*-1, are associated with resistance to the three main partner drugs: amodiaquine (AQ), lumefantrine and mefloquine. The 74I-75E-76T mutations of the *Pfcrt* gene and those of the *Pfmdr1* gene, N86Y-F184Y-D1246Y, are related to the parasite’s resistance to chloroquine (CQ) and AQ [15,16]. The N86-F184-D1246 haplotype of the *Pfmdr1* gene and the mutation detected at the E415G codon of the gene (exo-E415G) coding for exonuclease are associated with a decrease in parasite susceptibility to lumefantrine and piperaquine, respectively [17,18].

In Gabon, the treatment of uncomplicated malaria is based on the administration of AS-AQ and AL as first-line treatment and dihydroartemisinin-piperaquine phosphate (DHA-PQ) as second-line treatment. The injectable form of artesunate or quinine (if artesunate is unavailable) is recommended for the treatment of complicated malaria. Studies performed in different areas of this country reported a high frequency of molecular markers associated with resistance to aminoquinoline antimalarials and antifolates [19,20]. Otherwise, malaria prevalence in Gabon, after a drop between 2005 and 2011, has increased again in patients aged less than 20 years: 25% in 2011 and 36.3% in 2014 [21]. The prevalence of *P. falciparum* malaria was 32.8% among 1962 patients received in 2021 in the sentinel site for malaria surveillance of Melen (unpublished data). Children under 5 years old and those over 5 years old accounted for 68.9% and 54.5%, respectively (unpublished data). Among those aged over 5 years, 45.4% concerned children aged over 11 years (unpublished data). Likewise, the prevalence of severe malaria increased and a change in the clinical-biological profile was observed, with neurological forms being predominant [22].

Thus, the aim of this study was to analyze the relationship between the frequency of drug resistance molecular markers to ACTs and clinical forms of *P. falciparum* malaria according to parasite density level.

## 2. Materials and Methods

### 2.1. Study Area

A cross-sectional study was conducted between January and April 2014 in the Operational and Clinical Research Unit (OCRU), located in the Regional Hospital of Melen (RHM) in the north of Libreville. The RHM is located 11 km north of Libreville, as previously described [21]. Its equatorial climate is subdivided in four seasons: a short dry season from December to January, a long dry season from May to September, a short rainy season from October to November and a long rainy season from February to June. The malaria transmission is perennial in the country. *P. falciparum* is the cause of 99% of the symptomatic infections [20]. The major vector species are *Anopheles (A.) gambiae* and *A. funestus* [23]. 

### 2.2. Study Population

The participants were children aged 12 to 240 months mainly living in the district surrounding the RHM. Children meeting the following criteria were invited to participate: being febrile (with a tympanic temperature of >37.5 °C) or having a 24 h to 48 h history of fever before the day of consultation. A clinical examination was performed to identify the criteria of complicated malaria, looking for repeated convulsions, iterative vomiting and icterus in particular. Complicated malaria was defined and classified according to WHO indications [24]. Biological analyses were also performed for each patient. Indeed, health workers appointed in pediatric wards directed all patients towards the OCRU to benefit from the blood smear and cell blood count tests. Those who had a *P. falciparum* mono infection and parasite density of >2000 trophozoïtes per microliter of blood (p/µL) were included. Non-inclusion criteria were the presence of a mixed *Plasmodium* infection and non-*falciparum* infection. 

Approximately 3 mL of blood sample in a EDTA tube were used for the malaria diagnoses.

### 2.3. Malaria Diagnosis

Giemsa-stained thick and thin blood smears of peripheral blood were taken for each patient, as previously described [25]. A smear was only considered negative if no malaria parasites were seen in 100 microscopic fields using immersion oil. The thin blood smear made it possible to identify *Plasmodium* species. All patients infected with *Plasmodium* species were treated according to national recommendations. 

### 2.4. Leukocyte Depletion

Leukocyte depletion consists of the elimination of blood leukocytes before malaria parasite DNA extraction using CF11 columns containing cellulose powder. It reduces the amount of human DNA (contained in the nucleus of leukocytes) in a *Plasmodium* infected blood sample to obtain *P. falciparum* purified DNA. Leucocyte depletion was carried out at the Department of Parasitology-Mycology of the Faculty of Medicine. Venous blood sample from each patient was depleted of leucocytes within 6 hours following the sample collection. After the depletion of the leucocytes, a quality control of the sample was done based on microscopy. 

### 2.5. Molecular Analysis

Parasite genomic DNA was then extracted from samples after leucocytes depletion using a commercial Kit QIAamp DNA Blood Midi (100), according to the manufacturer’s instructions. DNA was kept in 100 µL of elution buffer at −80 °C for long-term conservation. The genes analyzed are reported in Table 1.

The NGS was carried out at the Wellcome Trust Sanger Institute (United Kingdom) for the detection of mutations related to resistance to ART and quinolines. To characterize ART, the resistance genes designated parasite genetic background (PGB) were sequenced (Table 1). Polymorphism on the *Pfcrt* (at codons 72–76) and *Pfmdr1* (at codons 86, 184 and 1246) genes was also analyzed in order to characterize the genetic profile of *Plasmodium* strains resistant to quinolines. All single nucleotide polymorphisms (SNPs) were further assessed using the 3D7 strain as a reference genome. 

This work was carried out as part of a multicenter study by different teams in 10 African countries for *PfK13* gene polymorphism analysis [30]. These teams gathered in the "Plasmodium Diversity Network African (PDNA)" network, aiming to study the genetic diversity of *P. falciparum*. This includes the study of molecular markers associated with resistance to artemisinin and partner molecules in *P. falciparum* isolates [26]. This network has established a collaboration with the Wellcome Trust Sanger Institute in order to generate standardized data.

#### 2.5.1. Genetic Report Card User Guide

The Genetic Report Card (GRC) contains malaria parasite genetic data, derived from the analysis of patient blood samples. For each sample, we determined the genotype for several variations (single nucleotide polymorphisms, or SNPs) that are known to be associated with resistance to antimalarials. The report also contains data on *Plasmodium* species found in the sample. The following is a guide to interpreting the Genetic Report Card data, linking the genotyped mutations (molecular markers) with resistance to different antimalarial drugs (Table A1 and Table A2).

#### 2.5.2. Drug Resistance Genes and Haplotypes

Several columns in the GRC show the genotypes of drug resistance mutations. For consistency with the published literature, these genotypes are reported as amino acid positions and alleles. For genes that carry multiple SNPs of interest, we concatenated the genotypes to form a haplotype, which is also common in the literature. In the haplotypes, we also used the following special characters:

A dash (“-”) indicates a missing genotype, either because the sample could not be genotyped, or because the assay is still under development/refinement. An asterisk (“*”) indicates that two alleles were detected (a heterozygous call) (Table A2).

#### 2.5.3. Sample Barcodes

The sample barcodes are formed by concatenated genotypes at 101 SNPs across the *P. falciparum* genome. These SNPs are all biallelic, i.e., only two alleles are observed. They were chosen for their usefulness in analyses of relationship between parasites, and are not associated with drug resistance. The full list of SNPs used is given in Appendix A. SNPs within the barcode are represented by the observed nucleotide (A, T, C and G). If the genotype is missing (could not be detected), the symbol “X” is used; the symbol “N” indicates that both alleles were observed (heterozygous call).

#### 2.5.4. Species Co-Infections

We detected the presence of different *Plasmodium* species by testing an SNP in the parasite mitochondria (PfMIT: 270) that carries different alleles in three different parasite species: *P. falciparum*, *P. vivax* and *P. knowlesi*. This assay has not yet been published but has been validated with quantitative PCR assays for detecting *P. vivax* and *P. falciparum*. 

#### 2.5.5. Artemisinin Drug Resistance

Parasite genetic background (PGB) mutations showed evidence of a genetic background of mutations that allowed for the emergence of *PfK13* mutations [11]. These mutations are: V127M in the *Pfarp-s10* (PF3D7_1460900) protein, D193Y in ferredoxin (PF3D7_1318100), N326S and I356T in *Pfcrt* (PF3D7_0709000) and T484I in *Pfmdr2* (PF3D7_1447900). These are displayed in a concatenated haplotype form, with the reference allele (WT) being VDNIT.

#### 2.5.6. Chloroquine Drug Resistance

Chloroquine drug resistance is primarily mediated by mutations in the chloroquine resistance transporter (*Pfcrt,* PF3D7_0709000) [31]. An accessory mutation at position 86 in the multidrug resistance protein (*Pfmdr1,* PF3D7_0523000) has been shown to accentuate this resistance phenotype in parasites [32]. The loci in *Pfcrt* are represented as a 5-amino acid haplotype at positions 72–76, with the wild-type haplotype being CVMNK [29]. The CVIET haplotype is the most widespread resistant haplotype in Asia and Africa, while SVMNT is common in resistant parasites in South America and Oceania. The *Pfmdr1* mutation at position 86 is the first in the three amino-acid haplotypes reported for this gene. The WT variant is N, while the Y variant in MDR-1 86 enhances resistance to chloroquine.

#### 2.5.7. Amodiaquine Drug Resistance

Mutations in the multidrug resistance protein (*Pfmdr1*, PF3D7_0523000) have been associated with parasite response to amodiaquine. There is limited evidence that mutations at positions 86 and 1246 can mediate the response to this drug, and these positions constitute the haplotype reported for this gene [33]. In vitro experiments have shown that haplotypes containing the mutant 86Y have increased IC50’s to chloroquine and amodiaquine.

#### 2.5.8. Mefloquine and Lumefantrine Drug Resistance

Mutations in the multidrug resistance protein (*Pfmdr1,* PF3D7_0523000) have been associated with parasite response to the drugs mefloquine and lumefantrine [28]. There is limited evidence that variations at positions 86, 184 and 1246 increase susceptibility to these drugs [28].

#### 2.5.9. Piperaquine Drug Resistance

In a recent genome-wide association study (GWAS), an SNP in a putative exonuclease gene (PF3D7_1362500) was associated with ex vivo piperaquine IC50 of parasite isolates from Cambodia [17]. This molecular marker is at position 415, and a G allele was shown to be significantly associated with increased tolerance of piperaquine with respect to the WT allele E.

### 2.6. Ethical Considerations

In Gabon, the Gabonese Ministry of Health, represented by the Malaria National Control Program, has committed the Department of Parasitology-Mycology and Tropical Medicine of the Faculty of Medicine of Libreville to perform an evaluation of malaria prevalence and antimalarial drug resistance monitoring in sentinel sites throughout the country. In this study, all patients were enrolled in the sentinel site for malaria surveillance in the RHM (Melen). In this context, patients and the guardian or parent of the children included were informed of the activities in this sentinel site and their signed consent was obtained. Children over the age of 12 gave their consent to participate in the study. In addition, in this site, a standardized procedure on the conservation of blood aliquots of febrile patients in the biobank was established. Being in a sentinel site, the participants were treated according to national recommendations.

### 2.7. Statistical Analysis

Data were analyzed using Epi InfoTM7. All variables were compared using Chi 2 or the Fisher test to compare the exact proportions, and Student’s *t*-test and analysis of variance (ANOVA) or the Kruskal-Wallis test, as appropriate for continuous variables. *p*-value < 0.05 was considered statistically significant.

## 3. Results

### 3.1. Characteristics of the Study Population

**Figure 1:**
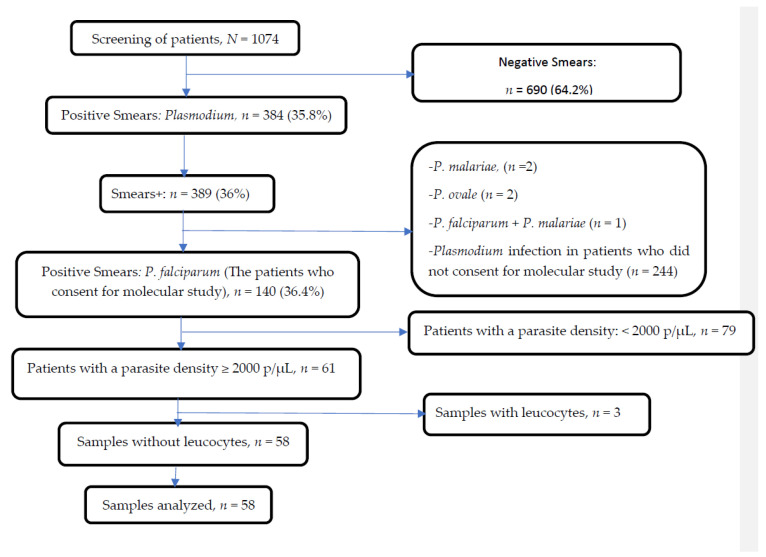
Profile of outcomes obtained before sequencing.

A total of 1074 patients were screened for malaria. Median age of the population was 69 (66–72) months, with a sex ratio of 1.23 (with 55.1% of patients of male sex). Among them, 384 had a *Plasmodium* infection. *P. falciparum* mono-infection was found in 98.9% of blood samples. Among those, sixty-one samples were selected for molecular studies (Figure 1).

Amplification and sequencing of the *Pfcrt*, *Pfmdr2*, *Pfarps10*, *Pffd* and *Pfmdr1* genes were successfully performed in 58 samples. These samples were from febrile patients, of whom 70.7% (n = 41/58) had uncomplicated malaria (UM) (Table 2). The median parasitemia was 32,200 (25,900–39,200) p/µL in clinical samples. The highest median parasite density was found in samples from patients with complicated malaria (CM) (*p* < 0.01) (Table 2). Hyperparasitemia (parasite density ≥200,000 p/µL) was found in six samples from patients with CM. Likewise, diarrhea, vomiting and convulsions were observed in two, three and six patients with CM, respectively. The use of antimalarial drugs before consultation applied to 17.6% of patients with CM and 82.3% of those with UM.

### 3.2. Artemisinin Resistance Related Molecular Markers Prevalence

Analysis of PGB *Pfarps10*, ferredoxin, *Pfcrt* and *Pfmdr2* genes showed that 37.9% of samples carried the mutant allele *Pfmdr2*-484I. The *Pfcrt*-326T mutant allele was found in all samples, while the mutant *Pfarps10*-127M and *Pffd*-193Y were not detected. The haplotypes identified were the double mutant VDNTI (27.5%, n = 16/58) and the single mutant VDNTT (62.0%, n = 36/58) (*p* < 0.01). Six isolates carried both haplotypes.

### 3.3. Piperaquine, Amodiaquine and Lumefantrine Resistance Related Molecular Markers Prevalence 

**Figure 2:**
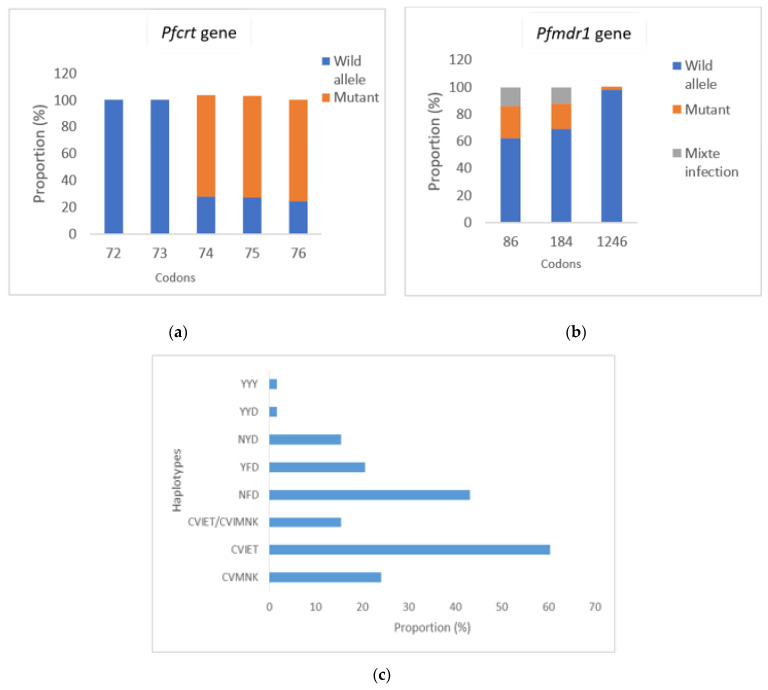
Frequency of molecular markers of amino-4-quinoleine resistance (*Pfcrt*-72-76 + *Pfmdr1*-86-184-1246). (**a**) Frequency of *Pfcrt* mutations related to amino-4-quinoleines resistance; (**b**) Frequency of *Pfmdr1* mutations related to AQ and lumefantrine resistance; (**c**) Frequency of haplotypes of *Pfcrt gene*, *Pfmdr1 gene* and *Pfcrt* + *Pfmdr1 genes*. YYY: 86Y-184Y-1246Y; YYD: 86Y-184Y-D1246; NYD: N86-184Y-D1246; YFD: 86Y-F184-D1246; NFD: N86-F184-D1246; CVMNK: 72C-73V-74M-75N-76K; CVIET: 72C-73V-74I-75E-76T; CVIET/CVMNK: mixed infection.

Exonuclease mutation E415G related to piperaquine resistance was not found in any isolates. Analysis of *Pfcrt* and *Pfmdr1* genes polymorphism showed that the *Pfmdr1*-86N allele and *Pfmdr1*-184Y allele were detected in 22 (37.9%) and 3 (5.2%) isolates, respectively. A single (1.72%) isolate had a *Pfmdr1*-1246Y mutant type allele. Three-quarters of the isolates had mutant alleles *Pfcrt*-76T, *Pfcrt*-74I and *Pfcrt*-75E (Figure 2a). No mutations were found at codon *Pfcrt*-72 and *Pfcrt*-73.

The *Pfcrt* mutant haplotypes CVIET and wild type CVMNK, based on codons 72–76, and the mutant *Pfmdr1* haplotypes YFD, NFD and wild type NYD, based on codons 86, 184 and 1246 were the main haplotypes detected (Figure 2c). The triple mutant haplotype CVIET was found in 60.4% of the samples. One sample contained a mixed infection (CVIET/CVMNK) (Figure 2c). The NFD single mutant allele was found more frequently than the double mutant haplotype YFD (43.1% vs. 20.7%) (*p* = 0.01) and the wild type haplotype NYD (43.2% vs. 15.5%) (*p* = 0.001).

### 3.4. Frequency of Haplotypes According to Malaria Clinical Forms

Considering the different haplotypes identified, the double mutant VDNTI haplotype was 1.4-fold (35.3% vs. 24.4%) more frequent in isolates from patients with CM, although the difference was not statistically significant (*p* = 0.3). Similarly, wild type haplotype NYD was almost twice as frequent in the isolates of these patients (23.5%; n = 4/17 vs. 12%; n = 5/41 in isolates of patients with uncomplicated malaria) (*p* = 0.4; OR 2.2154 [0.5146–9.5372]).

The haplotype CVMNK was found in the majority of the isolates of patients with uncomplicated malaria. In these, the proportion of the NFD haplotype tends to be more frequent: 58.8% (n = 10/17) vs. 48.7% (n = 20/41) (*p* = 0.5). Likewise, the double mutant allele YFD was twice as frequent (24.4%; n = 10/41 vs. 11.7%; n = 2/17 in isolates of patients with CM) (*p* = 0.4; OR 2.4194 (0.4700–12.4547)). 

The frequency of the triple mutant haplotype CVIET was comparable between patients with CM (58.8%; n = 10/17) and those with uncomplicated malaria (61%; n = 25/41). 

### 3.5. Haplotypes and Clinical and Biological Signs of Severe Malaria

Patients with hyperparasitemia or hyperparasitemia associated with convulsion were frequently infected by parasites with the CVIET triple mutant haplotype, VDNTT haplotype, NFD and YFD haplotypes of *Pfmdr1* gene. The VDNTI haplotype was frequent in isolates from patients with icterus and convulsions.

The combination of PGB and *Pfcrt* 72-76 haplotypes showed the presence of five haplotypes (H0 to H4) (Table 3). H0 and H2 were the most frequent (Table 3).

## 4. Discussion

In the present study, the monitoring of drug resistance molecular markers related to antimalarial drugs used in Gabon revealed mutations T484I (>37.8%) and I356T (100%) on the *Pfmdr2* and *Pfcrt* genes, respectively. In Asia, these are associated with a delayed clearance of parasites in patients treated with AL, DHA-PQ and AS-AQ, respectively [13]. The appearance of these mutations suggests that *P. falciparum* strains are under drug pressure. Thus, the occurrence of additional mutations on the *Pfarps* and *Pffd* genes may be expected, although none were found in these isolates. Similarly, molecular markers such as Exo-E415G SNP related to piperaquine resistance and DH-PQ treatment failure were not detected in the isolates. Nevertheless, *P. falciparum* strains carrying multiple copies of the *Plasmepsin 2* gene have been collected in previous studies carried out in Gabon, Burkina Faso, the Democratic Republic of Congo, Mozambique and Uganda [34]. Taken altogether, these data suggest a delayed appearance of the Exo-E415G mutation, compared to the *Plasmepsin 2* gene’s amplification in isolates circulating in areas where these molecules are used. Furthermore, the mutant *Pfmdr1*-86Y (43%) allele, which is less frequent than the mutant *Pfcrt*-76T allele (60%) in *P. falciparum* isolates from Melen was also detected. Its frequency confirms a decreasing frequency of this haplotype since the implementation of ACTs [19,35]. 

It is notable that multiple mutations are generally required to functionally confer and increase the level of drug resistance. The *Pfcrt* CVIET triple mutation was found in 60.4% of *P. falciparum* isolates from Melen. This proportion is similar to those reported in the Congo (71%) and south-east Gabon (70.6%) [36,37]. This haplotype was reported to be more frequently detected in isolates from West Africa (44%) and East Africa (38%), than in those from South Africa (21%) and Central Africa (26%) [38]. Among the *Pfmdr1* haplotypes, the NFD haplotype related to lumefantrine resistance was the most prevalent, found in almost one out of two isolates, several years after the introduction of AL and AS-AQ in Gabon. Similar frequencies, varying from 35% to 46%, were found in other areas of Gabon and in Tanzania [20,36]. In contrast, a higher prevalence of the NFD haplotype was found in Senegal (62.26%), Kenya (51%) and Ghana (60%) [38]. The circulation of *P. falciparum* strains carrying the NFD haplotype is frequently related to pressure due to the use of AL [20,39].

In addition to the use of antimalarial drugs as self-medication, as well as the non-observance of the recommended dosage, other factors, such as the parasite’s genetic background, may lead to an increase in the number of complicated malaria cases in Gabon [22].

In the present study, specific haplotypes varied according to malaria severity, as well as clinical and biological signs. Haplotypes NFD, YFD and CVMNK were more common in isolates from patients with uncomplicated malaria. In contrast, in those from patients with severe malaria, NYD and VDNTI were the most frequent, although these differences were not statistically significant. The CVIET haplotype was common in both groups of patients. The relationship between these haplotypes and the occurrence of severe malaria needs to be confirmed. Specific haplotypes have been identified, but no significant relationship between the clinical forms of malaria—or biological modifications and the frequency of drug resistance molecular markers—has been found. More specifically, patients with hyperparasitemia were infected by parasites with the CVIET and VDNTT haplotypes. The VDNTI haplotype was found in isolates from patients with icterus and convulsion. NFD and YFD were detected in parasites from patients with convulsion. A relationship between NYD and high parasitemia (*p* = 0.04) has been reported elsewhere [40]. In the current study, the combined haplotypes H2 (VDNTT + CVIET), H3 (VDNTT + CVIET/CVMNK) and H4 (VDNTT + CVIET/CVMNK) were found in patients infected with high parasite densities, suggesting their involvement in the parasite’s ability to multiply.

## 5. Conclusions

More than one decade after the withdrawal of CQ and the introduction of ACTs in Gabon, monitoring of drug resistance molecular markers remains critical to providing data on the propagation of artemisinin resistant parasites, as done in the present study. Moreover, the different genetic profiles found here and their variations according to the clinical and biological signs of severe malaria are additional arguments for their monitoring, and for an in-depth analysis of their role of the CIVIET haplotype of the *Pfcrt* gene on the fitness of the parasite.

## 6. Patents

This section is not mandatory but may be added if there are patents resulting from the work reported in this manuscript.

## Figures and Tables

**Table 1:** Molecular markers of antimalarial resistance.

Protein	Antimalarial	Gene	Amino-Acid Position	Wild	Mutant	References
PGB Artemisinin genetic background	ART AS AL DHA	*Pfarps10* *Ferredoxine* *Pfcrt* *Pfmdr2*	127 193 326 356 484	V D N I T	M Y S T I	[11] [26] [12]
EXO	PQ	*Exonuclease*	415	E		[17]
MDR1	CQ	*Pfmdr1*	86	N	Y	[27] [28]
	AQ		184	F	Y	
	LUM		1246	D	Y	
	MQ					
CRT	CQ AQ	*Pfcrt*	72	C	S	[29]
			73	V	M	
			74	M	I	
			75	N	E	
			76	K	T	

CQ: Chloroquine, AQ: amodiaquine, LUM: lumefantrine, DHA: dihydroartemisinin.

**Table 2:** Characteristics of the study population.

Characteristics	All Participants N = 58	Uncomplicated Malaria N = 41	Complicated Malaria N = 17	*p*-Value
**Median age, months**	69 (66–72)	48 (40–60)	60 (36–66)	<0.01
**Gender (Male/Female) ratio**	1.23	0.48	2.6	<0.01
**Median parasite density (µ/L)**	32,200 (25,900–39,200)	21,000 (19,600–25,200)	105,700 (87,500–156,100)	<0.01
**Mean T °C (±SD)**	37.5 ± 0.77	37.8 ± 0.76	39.5 ± 0.85	0.02

T °C: temperature in degrees Celsius, SD, standard deviation.

**Table 3:** Genetic profile of *P. falciparum* isolates according to median parasite density.

	PGB (Aminos Acids)	*Pfcrt* (Aminos Acids)	Total N = 58 n(%)	Median PD (25–75%)
**Haplotype 0 (H0)**	VDNTI	CVMNK	14 (24.2)	9100 (7700–10,500)
**Haplotype 1 (H1)**	VDNTI	CVIET	2 (3.4)	87,675 (4900–170,450)
**Haplotype 2 (H2)**	VDNTT	CVIET	34 (58.6)	39,900 (39,200–40,600)
**Haplotype 3 (H3)**	VDNTT/I *	CVIET, CVMNK	5 (8.6)	67,200 (42,000–133,000)
**Haplotype 4 (H4)**	VDNTT	CVIET, CVMNK	3 (5.2)	49,000 (9100–101,500)

PD: parasite density; *Pfarps10-*V127M-*Pffd-*D193Y- *Pfcrt*-N326-*Pfcrt-*I356T-*Pfmdr2-*T484I (VDNTT/ VDNTI); * Mixed infections at codon 484T/I of *Pfmdr2* gene; H0 is a combination of VDNTI and CVMNK; H1 is a combination of VDNTI and CVIET; H2 is a combination of VDNTT and CVIET; H3 is a combination of VDNTT * (* Mixed infection on 484T/I codon of *Pfmdr2* gene), CVIET and CVMNK); H4 is a combination of VDNTI, CVIET and CVMNK: The underline indicate the mutant allele.

## Data Availability

Not applicable.

## Appendix A

**Table A1:** List of barcode SNPs.

Multiplex	Chromosome	Position	Mutation	Multiplex	Chromosome	Position	Mutation
W36960	2	376222	K1929E	W36962	7	704373	389E
W36960	2	470013	G75E	W36962	7	1066698	G483S
W36960	3	656861	129V	W36962	7	1213486	S543N
W36960	4	110442	G285E	W36962	8	339406	1283C
W36960	4	881571	1081R	W36962	8	701557	394G
W36960	5	350933	1369N	W36962	8	1313202	799F
W36960	5	369740	907L	W36962	9	452690	1018I
W36960	6	900278	P696S	W36962	9	599655	E654D
W36960	7	1044052	686K	W36962	10	1383789	N114H
W36960	8	413067	1044V	W36962	10	1385894	815P
W36960	8	1314831	1342K	W36962	11	1006911	D124E
W36960	9	900277	1534E	W36962	11	1295068	E405K
W36960	11	1018899	1199L	W36962	11	1802201	450S
W36960	11	1815412	E765Q	W36962	12	858501	Q469K
W36960	13	1056452	1234D	W36962	12	1667593	2381N
W36960	13	1466422	N66K	W36962	12	1934745	241L
W36960	14	137622	1179V	W36962	13	159086	21R
W36960	14	2164225	2830S	W36962	13	388365	S1236R
W36961	1	145515	294I	W36962	13	1419519	Q208R
W36961	3	548178	R2L	W36962	13	2161975	D252V
W36961	4	139051	K438N	W36962	13	2573828	I1153M
W36961	4	286542	H586N	W36962	14	438592	N348T
W36961	4	529500	1477Y	W36962	14	2625887	M238I
W36961	4	1102392	E808D	W36962	14	3126219	S628F
W36961	5	796714	396K	W36963	1	179347	311G
W36961	7	461139	M361I	W36963	1	180554	D714N
W36961	7	619957	675R	W36963	1	283144	H664D
W36961	7	1256331	L321F	W36963	1	535211	2521F
W36961	8	417335	R244K	W36963	2	839620	260L
W36961	8	417335	R244K	W36963	4	426436	D560A
W36961	10	317581	311I	W36963	4	531138	A992E
W36961	10	336274	I1677V	W36963	4	891732	R4468S
W36961	11	477922	H147Y	W36963	5	172801	E218K
W36961	11	1020397	G700E	W36963	6	574938	I2934L
W36961	11	1294107	84A	W36963	7	635985	T598A
W36961	11	1935227	R73S	W36963	7	1308383	G1945R
W36961	12	1663492	1014E	W36963	7	1358910	K286E
W36961	12	2171901	V140D	W36963	7	1359218	K388N
W36961	13	1233218	N277S	W36963	8	150033	1315I
W36961	13	1867630	M4911I	W36963	8	399774	421K
W36961	13	2377887	2002S	W36963	8	1056829	L474I
W36961	14	2355751	H1589Q	W36963	9	1379145	R398Q
W36961	14	3046108	417V	W36963	10	1386850	927K
W36962	2	529709	F487L	W36963	11	408668	1058I
W36962	2	714480	D258G	W36963	11	828596	K240E
W36962	3	155697	150P	W36963	11	1935031	I139L
W36962	4	648101	51V	W36963	12	857245	E50G
W36962	4	1037656	2776I	W36963	14	107014	215K
W36962	5	1204155	1338I	W36963	14	1757603	D1365G
W36962	6	1282691	803K	W36963	14	2733656	557C
W36962	6	1289212	125T				

**Table A2:** Data base after sequencing.

ID	Species	CRT	MDR1	PGB	EXO
QW0009-CW	Pf	CVMNK	NYD	VDNTI	E
QW0010-CW	Pf	CVIET	YYY	VDNTT	E
QW0010-CxW	Pf	CVIET	YFD	VDNTT	E
QW0014-CW	Pf	CVMNK	NYD	VDNTI	E
QW0016-CW	Pf	CVMNK	NYD	VDNTI	E
QW0018-CW	Pf	CVIET,CVMNK	**D	VDNT*	E
QW0019-CW	Pf	CVIET,CVMNK	NYD	VDNT*	E
QW0020-CW	Pf	CVMNK	NFD	VDNTI	E
QW0021-CW	Pf	CVIET	NFD	VDNTT	E
QW0022-CW	Pf	CVIET	NYD	VDNTT	E
QW0023-CW	Pf	CVIET	NFD	VDNTT	E
QW0025-CW	Pf	CVIET	YFD	VDNTT	E
QW0027-CW	Pf	CVIET	YYD	VDNTT	E
QW0031-CW	Pf	CVIET,CVMNK	YFD	VDNT*	E
QW0033-CW	Pf	CVIET	NFD	VDNTT	E
QW0035-CW	Pf	CVIET	**D	VDNTT	E
QW0038-CW	Pf	CVMNK	YFD	VDNTI	E
QW0041-CW	Pf	CVIET	**D	VDNTT	E
QW0043-CW	Pf	CVIET	NFD	VDNTT	E
QW0046-CW	Pf	CVIET	NFD	VDNTT	E
QW0049-CW	Pf	CVIET	YFD	VDNTT	E
QW0050-CW	Pf	CVIET	YFD	VDNTT	E
QW0057-CW	Pf	CVMNK	NYD	VDNTI	E
QW0058-CW	Pf	CVIET	NFD	VDNTI	E
QW0064-CW	Pf	CVIET	NFD	VDNTT	E
QW0067-CW	Pf	CVMNK	NFD	VDNTI	E
QW0071-CW	Pf	CVIET,CVMNK	*FD	VDNT*	E
QW0072-CW	Pf	CVIET	N*D	VDNTI	E
QW0074-CW	Pf	CVIET	NFD	VDNTT	E
QW0076-CxW	Pf	CVIET	NFD	VDNTT	E
QW0077-CW	-	-	---	-----	-
QW0077-CxW	Pf	CVIET	NFD	VDNTT	E
QW0081-CW	Pf	CVIET	N*D	VDNTT	E
QW0083-CxW	Pf	CVIET	NFD	VDNTT	E
QW0084-CxW	Pf	CVIET	YFD	VDNTT	E
QW0087-CW	Pf	CVIET	**D	VDNTT	E
QW0088-CW	Pf	CVMNK	YFD	VDNTI	E
QW0089-CxW	Pf	CVIET	NFD	VDNTT	E
QW0090-CW	Pf	CVIET	NFD	VDNTT	E
QW0090-CxW	Pf	CVIET	NFD	VDNTT	E
QW0091-CxW	Pf	CVIET,CVMNK	**D	VDNTT	E
QW0092-CxW	Pf	CVIET	NFD	VDNTT	E
QW0096-CW	Pf	CVIET	NFD	VDNTT	E
QW0100-CW	Pf	CVMNK	NFD	VDNTI	E
QW0101-CW	Pf	CVIET	YFD	VDNTT	E
QW0107-CW	Pf	CVMNK	NYD	VDNTI	E
QW0109-CW	Pf	CVIET	NFD	VDNTT	E
QW0115-CW	Pf	CVMNK	NFD	VDNTI	E
QW0118-CW	Pf	CVMNK	YFD	VDNTI	E
QW0119-CxW	Pf	CVIET	NYD	VDNTT	E
QW0120-CxW	Pf	CVMNK,CVIET	NYD	VDNT*	E
QW0125-CxW	Pf	CVIET	YFD	VDNTT	E
QW0127-CW	Pf	CVIET,CVMNK	*FD	VDNTT	E
QW0127-CxW	Pf	CVIET,CVMNK	YFD	VDNTT	E
QW0129-CW	Pf	CVIET	NFD	VDNTT	E
QW0139-CxW	Pf	CVIET	*FD	VDNTT	E
QW0144-CxW	Pf	CVMNK	NFD	VDNTI	E
QW0146-CW	Pf	CVMNK	NFD	VDNTI	E
QW0148-CW	Pf	CVMNK,CVIET	NFD	VDNT*	E
